# Network Analysis of Linkage Disequilibrium Reveals Genome Architecture in Chum Salmon

**DOI:** 10.1534/g3.119.400972

**Published:** 2020-03-12

**Authors:** Garrett McKinney, Megan V. McPhee, Carita Pascal, James E. Seeb, Lisa W. Seeb

**Affiliations:** * College of Fisheries and Ocean Sciences, University of Alaska Fairbanks, 17101 Point Lena Loop Road, Juneau, AK, 99801; † School of Aquatic and Fishery Sciences, University of Washington, 1122 NE Boat Street, Box 355020, Seattle WA 98195

**Keywords:** chum salmon, inversion, x-chromosome, linkage disequilibrium, network analysis

## Abstract

Many studies exclude loci that exhibit linkage disequilibrium (LD); however, high LD can signal reduced recombination around genomic features such as chromosome inversions or sex-determining regions. Chromosome inversions and sex-determining regions are often involved in adaptation, allowing for the inheritance of co-adapted gene complexes and for the resolution of sexually antagonistic selection through sex-specific partitioning of genetic variants. Genomic features such as these can escape detection when loci with LD are removed; in addition, failing to account for these features can introduce bias to analyses. We examined patterns of LD using network analysis to identify an overlapping chromosome inversion and sex-determining region in chum salmon. The signal of the inversion was strong enough to show up as false population substructure when the entire dataset was analyzed, while the effect of the sex-determining region on population structure was only obvious after restricting analysis to the sex chromosome. Understanding the extent and geographic distribution of inversions is now a critically important part of genetic analyses of natural populations. Our results highlight the importance of analyzing and understanding patterns of LD in genomic dataset and the perils of excluding or ignoring loci exhibiting LD. Blindly excluding loci in LD would have prevented detection of the sex-determining region and chromosome inversion while failing to understand the genomic features leading to high-LD could have resulted in false interpretations of population structure.

The vast amount of genomic data now available allows research to move beyond analysis of allele frequency distributions among populations and into the effects of genomic structure and organization on adaptation and population structure. Genomic research has illuminated a range of evolutionary processes influencing adaptation. Single gene differences have been shown to have an important role in adaptation and life-history variation: see for example the *Eda* gene that influences armor plating in stickleback (Schluter *et al.* 2010) and the *VGLL3* gene that maintains variation in age at maturity in Atlantic salmon (*Salmo salar*) (Barson *et al.* 2015). On a larger genomic scale, the importance of islands of divergence (*e.g.*, Larson *et al.* 2017) and chromosome inversions (Wellenreuther and Bernatchez 2018) is increasingly recognized in non-model organisms, particularly in the context of sexually antagonistic selection (Kirkpatrick and Guerrero 2014; Barson *et al.* 2015). While islands of divergence can exhibit elevated linkage disequilibrium (LD) due to genetic hitchhiking, other features of genomic architecture such as inversions or sex-determining regions inhibit recombination, resulting in elevated LD and the inheritance of haplotype blocks. Historically, many studies explicitly excluded loci exhibiting LD under the assumption that these markers provide redundant information (*e.g.*, Larson *et al.* 2014), but with the current recognition of their importance and ability to detect genomic architecture, that practice is becoming obsolete.

Chromosome inversions have been associated with widely divergent adaptive variation ranging from social behavior (Thomas *et al.* 2008), life-history variation (Miller *et al.* 2012), alternative reproductive strategies (Küpper *et al.* 2015), and adaptation to different environments (Jones *et al.* 2012). Inversions have been implicated in the formation and divergence of sex chromosomes (Lemaitre *et al.* 2009) and establishment of reproductive barriers leading to speciation (Noor *et al.* 2001). Inversions often exhibit elevated divergence due to inhibited recombination, which can manifest as population substructure in genetic analyses (Tian *et al.* 2008; Arostegui *et al.* 2019), and the extended LD exhibited by large inversions facilitates their detection in marker-dense next-generation sequencing projects (Kemppainen *et al.* 2015). An understanding of the extent and geographic distribution of inversions is now a critically important part of genetic analyses of natural populations.

Sex chromosomes are another genomic feature that often exhibits reduced recombination and elevated LD, with important evolutionary implications as males and females often exhibit different phenotypes and experience different selective pressures (Badyaev and Hill 2000; Tamate and Maekawa 2006). Elevated LD in sex chromosomes can be due to the presence of inversions (Lemaitre *et al.* 2009; Connallon *et al.* 2018) or as a result of sex-specific patterns of recombination (Kijas *et al.* 2018). Reduced recombination can facilitate divergence between sex chromosomes under sexually antagonistic selection (Charlesworth 2018). Sexually antagonistic selection has been noted in a number of cases such as ornamental traits in poeciliid fishes (Lindholm and Breden 2002), genetic variation for fitness in red deer (Foerster *et al.* 2007), and optimal age at maturity in Atlantic salmon (Barson *et al.* 2015). When causal variants are located on the sex chromosome, sexually antagonistic selection can be resolved through portioning of genetic variation between sexes (Roberts *et al.* 2009).

With increasingly dense genomic data, sex-associated loci are likely to be found even if the sex-chromosome has not been identified; but, when unaccounted for, these sex-associated markers can bias genomic analyses (*e.g.*, Benestan *et al.* 2017). Traditionally, sex-associated markers have been identified through genome-wide association studies (GWAS) of individuals with known sex or by identifying characteristics of heterogametic sex loci, such as presence/absence or genotypic frequencies where half of the samples are heterozygous and the other half are a single homozygous genotype (*e.g.*, Star *et al.* 2016; McKinney *et al.* 2020). An alternative approach is to search for regions of extended LD which are often associated with sex-determining regions.

Identification of genomic features should be a routine step in genomic analyses to avoid biases outlined above (Benestan *et al.* 2017; Arostegui *et al.* 2019). Genome assemblies allow direct visualization of LD patterns along the chromosome, but most species do not have assembled genomes. In this case, network analysis can be performed on patterns of LD to identify genomic features (Kemppainen *et al.* 2015). Network analysis is likely to be particularly informative when multiple genomic features overlap, manifesting as a single region of high LD.

Here, we describe the detection and genetic architecture of multiple genomic features in chum salmon (*Oncorhynchus keta*) from western Alaska. Chum salmon exhibit an anadromous life history where individuals emerge in freshwater and then migrate to the ocean to mature before returning to freshwater streams to spawn. Populations often exhibit life-history variation in the timing of return migration. We used patterns of LD combined with network analysis to discover a chromosomal inversion in chum salmon that contains the sex-determining region. The inversion exhibits spatial variation in frequency throughout western Alaska and includes putatively adaptive genes associated with life-history variation in other salmonids.

## Materials and Methods

### SNP discovery and RAD sequencing

**Figure 1:**
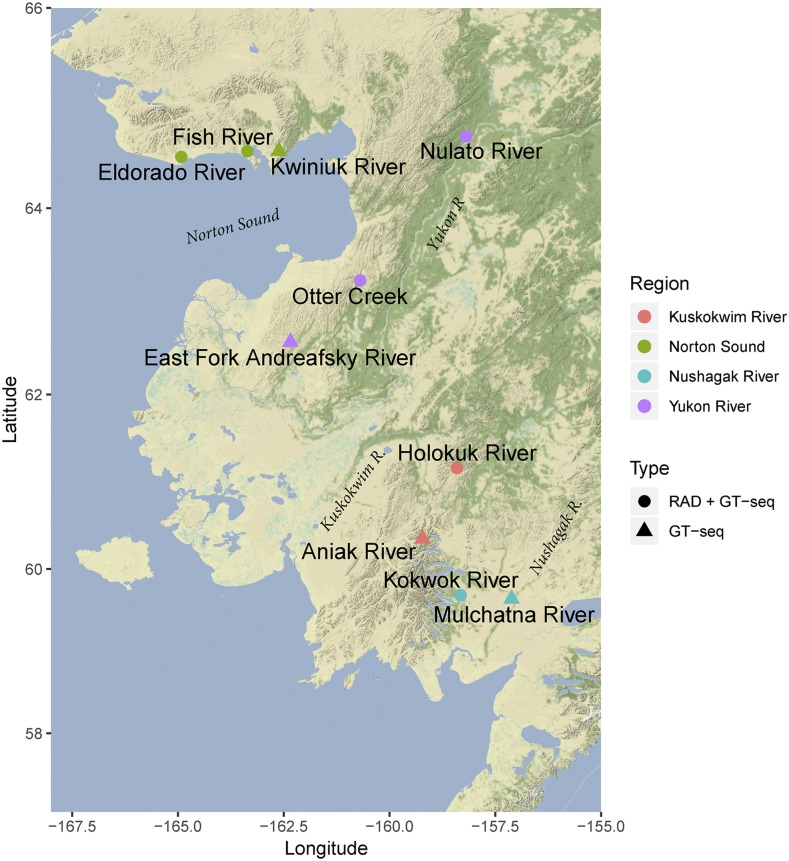
Map of sampling locations. Collections are colored by region, and shapes denote whether samples were genotyped using RADseq and GT-seq or GT-seq only.

SNP discovery was conducted on 6 collections of chum salmon (48 samples each) using RAD sequencing (Figure 1). These collections were distributed among four major regions: Norton Sound, Yukon River, Kuskokwim River, and Nushagak River (Table 1). DNA was obtained from Alaska Department of Fish and Game (ADFG).

**Table 1:** Number of samples initially sequenced and retained after quality filtering for RADseq and GT-seq datasets. Collections used to evaluate accuracy of the putative sex- determining region are marked with an asterisk *

Region	Collection	Initial RADseq Samples	Retained RADseq Samples	Initial GT-seq Samples	Retained GT-seq Samples
Norton Sound	Eldorado River	48	48	83	82
Norton Sound	Fish River*	48	39	82	77
Norton Sound	Kwiniuk River*	0	0	82	74
Yukon River	Nulato River	48	38	83	80
Yukon River	Otter Creek	48	48	99	92
Yukon River	East Fork Andreafsky River	0	0	83	83
Kuskokwim River	Holokuk River	48	48	83	79
Kuksokwim River	Aniak River	0	0	82	79
Nushagak River	Kokwok River	48	46	83	79
Nushagak River	Mulchatna River*	0	0	82	73
		288	267	842	798

Sequencing libraries were prepared with the *SbfI* enzyme using a modification of the improved RADseq library preparation protocol developed in Ali *et al.* (2016). Samples were sequenced on a HiSeq 4000 with paired-end 150 bp reads; 96 samples were sequenced per lane. Two rounds of sequencing were conducted, with the volume of DNA for each individual adjusted in the second round of sequencing to reduce variation in sequence reads per individual (Prince *et al.* 2017). Sequence data were processed with *STACKS* v1.47 (Catchen *et al.* 2011) using default settings with the following exceptions: process_radtags (-c -r -q -t 140), ustacks (-r–model_type bounded–bound_low 0–bound_high 0.01), cstacks (-n 2). The catalog of variation for *STACKS* was created using six individuals from each collection.

Loci and samples were filtered using an iterative process where poor-quality loci and samples were removed initially, the proportion of missing data were recalculated, and more stringent thresholds were applied for final filtering. A genotype rate threshold of 50% was initially applied to remove poor-quality loci. The genotype rate per sample was then estimated using the retained loci; samples were retained if they had a genotype rate of at least 75%. This iterative process prevents filtering of good quality samples due to an excess of poor quality loci, as well as filtering of good quality loci due to an excess of poor quality samples. Allele frequencies and *F*_ST_ were estimated with *Genepop* (Rousset 2008) using the retained samples; loci with a minor allele frequency (MAF) of at least 0.05 were retained. *HDplot* (https://github.com/gjmckinney/HDplot, McKinney *et al.* 2017) was used to identify paralogs, which were excluded from further analysis because read depth was too low for accurate genotyping (McKinney *et al.* 2018). Singleton (non-paralogous) loci were retained for final analysis if they met a threshold of 90% genotype rate. Retained singleton loci were aligned against the rainbow trout *O. mykiss* genome (Omyk_1.0, NCBI: GCF_002163495.1) using *bowtie2* v2.2.1 (Langmead and Salzberg 2012) to allow investigation of genomic patterns of differentiation.

### Population structure

Genomic features can manifest as population substructure when reduced recombination leads to fixation of alternate alleles over large genomic areas (*e.g.*, Arostegui *et al.* 2019). Population structure was visualized using individual-based PCAs in R with Adegenet (Jombart 2008) to determine if any populations exhibited patterns of substructure. The populations included in this study spanned a large geographical region in Alaska, so population structure was examined within each region to prevent large-scale population structure from overwhelming any signal of genomic features. Populations exhibiting substructure were candidates for initial examination of LD.

### Identification of genomic structures

Genomic features were identified by examining LD between markers. Pairwise LD was estimated between SNPs using the r-squared method in *Plink* (v1.07, Purcell *et al.* 2007). Pairwise LD was plotted for each chromosome using R to identify regions of elevated LD. Network analysis and community detection were conducted in R using the igraph package (https://igraph.org/r) to identify groups of linked SNPs, hereafter referred to as ‘sets’. This was done to determine if multiple patterns of LD were present within a single genomic region. For network analysis, SNP pairs with an r^2^ < 0.3 were removed. SNPs that were in LD with fewer than 3 other SNPs were also excluded to reduce the number of SNPs that were linked only by close physical proximity. Following network analysis, chromosomal positions of SNPs in LD sets were examined to determine if they could be attributed to genomic structures. Finally, patterns of variation for SNPs within LD sets were visualized using PCA.

### High-throughput assay panel

Two putative, co-occurring genomic features were identified from the RADseq data (see results): a chromosome inversion and a sex-determining region.

GT-seq assays were developed for 39 loci diagnostic for the putative inversion and 21 loci diagnostic for the putative sex-determining region. Multiple loci for each genomic feature were included to provide a control for genotyping error and because some loci would be routinely lost during panel optimization (described below). Filters were applied prior to and during primer design to remove loci that were likely to amplify off-target sequence with GT-seq following the methods of McKinney *et al.* (2020); this included identifying transposable elements and primers that align to multiple genomic regions. Loci with SNPs within 20 bp of the end of the RAD tag required genomic sequence past the RAD tag for primer design. We obtained a draft copy of the chum salmon genome (B.F. Koop, University of Victoria, Victoria, British Columbia, Canada; personal communication) and aligned RAD tag sequences to the unassembled scaffolds using *bowtie2*; custom perl scripts were used to obtain flanking sequence for primer design. Primers were designed using *BatchPrimer3* (You *et al.* 2008). Amplicons for retained primers were then examined to ensure that SNPs were contained within the first 100 bp of the amplicon to facilitate downstream single-end sequencing.

Two rounds of panel optimization were conducted in order to remove loci that did not perform well. Each round of optimization was conducted using 48 individuals sequenced on a MiSeq with 150 bp paired-end sequence. DNA was extracted and sequencing libraries were prepared following the methods of Campbell *et al.* (2015). Sequencing data were processed and genotyped using *GTscore* (https://github.com/gjmckinney/GTscore, McKinney *et al.* 2020). After each round of sequencing, the number of reads amplified by each primer was counted, as well as the proportion of reads that contained both the primer and bioinformatics probes for a locus. Loci with excessive amplification and off-target amplification were removed following the methods of McKinney *et al.* (2020).

An expanded sample of individuals was genotyped using the GT-seq panel to better characterize genomic structure across the region. This included additional individuals from the ascertainment collections as well as additional collections from Norton Sound, and the Yukon, Kuskokwim, and Nushagak rivers (Table 1). Populations and individuals with paired sex data were preferentially chosen to assess concordance between phenotypic sex and the putative sex-determining region. Sequencing libraries were prepared as above. A total of 871 samples (842 plus 39 sequenced twice with GT-seq for quality control) were sequenced on a single lane of a HiSeq 4000 with 100 bp single-end sequencing. These samples were also genotyped for an additional 478 markers on this sequencing lane as part of a separate collaborative project (McKinney *et al.*, unpubl.). All GT-seq loci were used for evaluating sample quality even though loci developed for the separate project are not included in this study. Samples were evaluated for quality based on a minimum 90% genotype rate and visualization of allele scatter plots.

### Data availability

Raw RADseq is available in NCBI BioProject PRJNA611968. Raw GT-seq data are available in NCBI BioProject PRJNA609360.The authors affirm that all data necessary for confirming the conclusions of this article are represented fully within the article and its tables and figures. Supplemental material available at figshare: https://doi.org/10.25387/g3.11903409.

## Results

### SNP discovery and RAD sequencing

*STACKS* initially outputted 222,668 SNPs within 94,002 RAD tags. A total of 30,006 SNPs within 22,693 RAD tags were retained after applying all filters (Table S1). Of these loci, 13,015 SNPs within 10,821 RAD tags were aligned to the rainbow trout genome. After all filtering steps, 267 individuals were retained for SNP discovery.

### Identification of genomic structures

**Figure 2:**
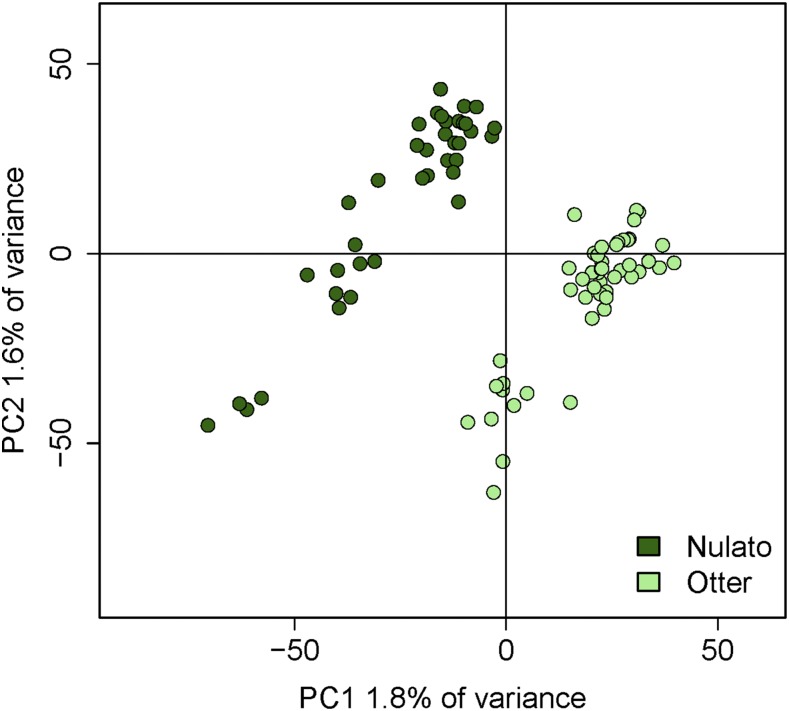
Individual PCA using all loci for Yukon River collections. Both collections exhibit patterns of substructure.

**Figure 3:**
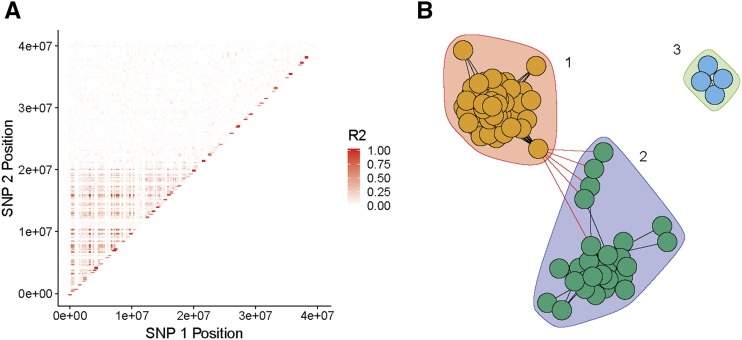
A) Plot of linkage disequilibrium (pairwise r^2^) of SNPs aligned to O. mykiss chromosome 28 (chum LG 15). Each point is a SNP pair colored by LD. The pattern of elevated LD spans 20 Mb of chromosome 28. B) Network analysis with community detection identified three distinct sets of loci contributing to LD on LG15. Set 1 (red background) has 51 loci, set 2 (purple background) has 27 loci, and set 3 (green background) has 4 loci. Loci in sets 1 and 2 span the entire 20 Mb while loci in set 3 are linked due to close physical proximity.

Clear patterns of population substructure were apparent in the Yukon River from individual-based PCAs using all 13,015 markers (Figure 2). No substructure was visible in individual PCAs for other geographic regions (Figure S1). The loci driving population substructure within Yukon River collections were aligned to a region of elevated LD on Omy28 (Figure 3A) which corresponds to linkage group 15 on the chum salmon linkage map (Waples *et al.* 2016; Sutherland *et al.* 2016). To avoid confusion, we refer to the chromosome as LG15 throughout even though SNP positions are derived from alignments to the *O. mykiss* chromosome 28. Network analysis identified three distinct sets of loci that exhibited high LD (Figure 3B). Loci in set 1 spanned from 437 Kb to 20.3 Mb, and the loci in set 2 spanned from 1.6 Mb to 21.3 Mb. The loci in set 3 spanned only a 2 Kb region, suggesting that their high LD is likely due only to close physical proximity. Therefore, these loci were excluded from further analysis.

**Figure 4:**
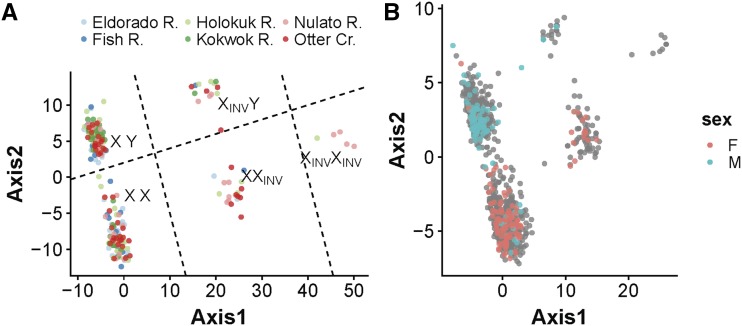
A) Individual PCA of RADseq samples using all loci from LG15. Examination of locus loadings show that Axis 1 is primarily driven by loci in set 1 (inversion loci) and axis 2 is primarily driven by loci in set 2 (sex-associated loci). Labels for each cluster of individuals denote the putative chromosome type of individuals within the cluster with respect to inversion and sex. B) Individual PCA of RADseq and GT-seq samples using loci successfully developed into GT-seq assays. Samples are color coded by phenotypic sex with gray individuals having unassigned sex.

We then plotted all RADseq samples for all loci from LG15. The PCA of LG15 revealed clustering of samples that was driven by the loci in sets 1 and 2; PCA loadings show that Axis 1 was primarily driven by loci in set 1, and axis 2 was primarily driven by loci in set 2 (Figure 4A).

**Figure 5:**
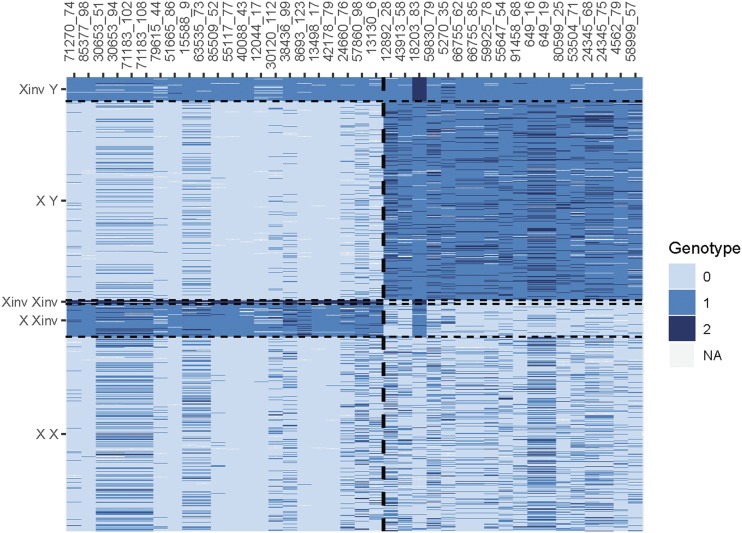
Genotypes for combined RADseq and GT-seq samples. Rows are samples ordered by sex (inferred from PCA cluster) and inversion type; columns are loci. Loci were separated by genomic feature to aid in visualization: inversion-associated loci are to the left of the dashed line and sex-associated loci are to the right of the dashed line. Within each genomic feature, loci are ordered by position. Individual genotypes are color coded with 0 and 2 representing alternate homozygous genotypes and 1 being a heterozygous genotype. Prefix of Oke_uwRAD was dropped from marker names for brevity.

Genotype patterns within a genomic feature can give clues to the type of genomic feature. Genotypes for loci in sets 1 and 2 (including those genotyped with GT-seq; see below) were plotted by SNP set and position to better visualize genotype patterns (Figure 5); this plot revealed patterns consistent with multiple genomic features within this region. Loci in set 1 exhibited three genotype classes (both homozygous and heterozygous) and near-complete linkage for loci spanning 20 Mb, which is consistent with a genomic inversion that is variable within populations (*e.g.*, Arostegui *et al.* 2019). While we could not directly test for an inversion using methods such as breakpoint mapping or linkage mapping, it is unlikely that the observed pattern of LD spanning 20Mb with three alternate genotypes could result from another process. We assume that the inversion was the least common form but this is not always the case (Arostegui *et al.* 2019). This inversion was present in all collections, but its frequency varied by region, with Yukon and Kuskokwim rivers having the highest frequency of the inversion (Table 2).

**Table 2:** Frequency of the chromosome inversion and sex-assignment accuracy by collection. Samples were assigned an inversion type (+/−) and sex based on PCA clustering. For collections with phenotypic sex data, phenotypes were compared to sex assigned through clustering to assess accuracy

Region	Collection	Data Source	++	+-	–	Freq (-)	M cluster	F cluster	Sex Assignment Accuracy
Norton Sound	Eldorado River	RAD/GT-seq	96%	4%	0%	2%	50	80	NA
Norton Sound	Fish River	RAD/GT-seq	90%	10%	0%	6%	54	62	84%
Norton Sound	Kwiniuk River	GT-seq	92%	8%	0%	4%	30	44	89%
Yukon R.	Nulato River	RAD/GT-seq	73%	23%	4%	22%	65	53	NA
Yukon R.	Otter Creek	RAD/GT-seq	76%	22%	1%	16%	65	75	NA
Yukon R.	East Fork Andreafsky	GT-seq	83%	17%	0%	10%	37	46	NA
Kuskokwim R.	Holokuk River	RAD/GT-seq	89%	10%	1%	7%	54	73	NA
Kuskokwim R.	Aniak River	GT-seq	85%	15%	0%	9%	41	38	NA
Nushagak R.	Kokwok River	RAD/GT-seq	96%	4%	0%	2%	96	29	NA
Nushagak R.	Mulchatna River	GT-seq	90%	10%	0%	5%	37	36	99%

Loci in set 2 exhibited a more complex pattern that is consistent with a sex-determining region. Putative males in the top half of Figure 5 and loci to the right of the dividing line show a pattern of very high heterozygosity with homozygous genotypes almost entirely of one class rather than both homozygous genotypes being equally represented. Putative females in the bottom half of Figure 5 with loci to the right of dividing line show a pattern of high homozygosity for the alternate allele found in males, and all three genotype classes are present. This overall pattern is consistent with fixation of one allele on the Y-chromosome with the alternate allele at high frequency, but not fixed, on the ancestral X-chromosome. These patterns are consistent with an XY sex-determining region and balanced sex ratios. This finding was further supported by phenotypic sex data (see below).

### High-throughput panel for screening genomic structures

Development of the GT-seq panel involved filtering steps during primer design and panel optimization. After evaluation and optimization, the final GT-seq panel contained 22 SNPs diagnostic for a putative inversion and 18 SNPs diagnostic for a putative sex-determining region. Primer sequences for final loci are located in Table S2 and bioinformatic probes used for genotyping in *GTscore* are in Table S3. A total of 43 individuals were removed for failing quality control: 19 samples had <90% genotype rate and an additional eight had broad allelic scatter due to reduced read depth (Figure S2B), five individuals showed elevated heterozygosity and indistinct allelic clusters suggesting contamination (Figure S2C), one individual showed four clusters consistent with triploidy (Figure S2D), two individuals showed five clusters consistent with tetraploidy (Figure S2E), and one individual had no heterozygous genotypes and was likely a different species of salmon (Figure S2F).

Inversion type and sex were assigned by clustering samples with PCA (Figure 4B); this PCA mirrored the results from RADseq analysis shown in Figure 4A. Results for inversion and sex are reported for the full dataset with the RADseq and GT-seq samples combined. Phenotypic sex data were available for samples in five of the collections examined; however, systemic errors in phenotypic records were identified for the East Fork Andreafsky and Aniak Rivers populations, causing phenotypes for these populations to be removed from analysis. The average concordance between phenotypic sex and cluster sex for the remaining combined RADseq and GT-seq datasets was 90% (range 84–99%, Table 2). Phenotypic sex of each individual, along with results of inversion type and sex assignment can be found in Table S4.

Similar to the RADseq data alone, within the full dataset the inversion was found in every collection but showed regional variation in frequency. Inversion frequency ranged from 2 to 22% and was most common in the Lower Yukon collections followed by the Kuskokwim River collections (Table 2). Outside of the Yukon and Kuskokwim rivers the inversion reached a maximum frequency of only 6% (Table 2).

## Discussion

Genomic structures including inversions and sex-determining regions provide important contributions to adaptive variation and population structure (Wellenreuther and Bernatchez 2018; Benestan *et al.* 2017). Large inversions are associated with adaptation and life- history variation across taxa, including ecotypes in mosquitos (*Anopheles* sp.) (Ayala *et al.* 2017), migration *vs.* residency in rainbow trout (*Oncorhynchus mykiss*) (Pearse *et al.* 2019), and annual *vs.* perennial yellow monkey flowers (*Mimulus guttatus*) (Lowry and Willis 2010). Inhibited recombination within inverted regions increases divergence between chromosomal forms and can drive patterns of population structure (Tian *et al.* 2008; Arostegui *et al.* 2019). A similar pattern has been observed for sex chromosomes where sex-associated markers can drive patterns of population structure (Benestan *et al.* 2017). We identified two genomic structures in chum–a chromosome inversion and sex-determining region–that occur in the same genomic region. The signal of the chromosome inversion was strong enough to cause population structuring when all markers were used for PCA; however, population structure due to the sex-determining region was only visible in the chromosome-specific PCA.

### Identification of genomic structures using LD

Population substructure shaped by the inversion and sex-determining region was only determined through a combination of LD and network analysis. Both the inversion and sex-determining region exhibited LD spanning the same genomic region, which would be difficult to ascertain manually. Network analysis and community detection was a simple method to automate detection of different groups of linked markers that contributed to this overall pattern of LD. Previous work has shown that a combination of LD and network analyses facilitates detection of genomic features even in the absence of reference genome (Kemppainen *et al.* 2015). Here we demonstrated that LD and network analysis can be used to tease apart multiple genomic features within the same chromosomal region.

The extended LD combined with the specific genotype patterns observed for high-LD loci suggests a genomic inversion that overlaps with the sex-determining region in chum salmon. The genotype patterns also suggest that the inversion arose on the X chromosome. An inversion arising on the X chromosome should result in 5 different chromosomal combinations: XX, XX_INV_, X_INV_X_INV_, XY, X_INV_Y. This agrees with the observed pattern of 5 sample clusters on the individual PCA (Figure 4). If the inversion had arisen on the Y chromosome only three clusters would be expected: XX, XY, XY_INV_. Levels of LD for sex-associated loci varied depending on the X-chromosome type, suggesting different levels of divergence between the Y-chromosome and the ancestral and inverted X-chromosomes.

Salmonids exhibit sex-specific recombination patterns where females recombine across the full length of chromosomes while recombination in males is restricted to telomeric regions (Lien *et al.* 2011; Pearse *et al.* 2019). While autosomes will be free to recombine in females, recombination between the X and Y can only take place in males and will be limited to telomeric regions. This type of recombination will likely result in patterns of extended LD as the sex chromosomes diverge due to genetic drift. Extended LD on sex-chromosomes has also been noted in Atlantic salmon (Kijas *et al.* 2018) and Chinook salmon (McKinney *et al.* 2019 preprint) and may be a general feature in salmonids.

### Chromosome inversion

An inversion on the X chromosome containing the sex-determining region has interesting evolutionary implications. Females can exhibit all three genotypes of the inversion, while males can be homozygous for the ancestral form or heterozygous for the inversion but never homozygous for the inversion. Inversions can lead to fixation of genetic variants on alternate chromosomes and facilitate adaptive divergence (reviewed in Wellenreuther and Bernatchez 2018). In salmonids, an inversion on Omy05 is associated with variation in migrant *vs.* resident life history in rainbow trout (Miller *et al.* 2012; Pearse *et al.* 2014; Pearse *et al.* 2019; Arostegui *et al.* 2019) . We cannot determine if the inversion we identified in chum salmon is adaptively important with the data currently available, but one intriguing result is that the inversion contains the *Greb1L* gene. This gene has been associated with variation in the timing of migration return in both Chinook salmon and rainbow trout (Prince *et al.* 2017; Micheletti *et al.* 2018; Hess *et al.* 2016).

Chum salmon exhibit two ecotypes that vary in season of migration into freshwater: summer migrants and fall migrants. In the Yukon River, summer chum salmon enter the river between early June and early July and spawn primarily in tributaries in the lower 700 km of the river. Fall chum salmon, the less-abundant ecotype, enter in mid-July to late August and, unlike the majority the chum salmon populations throughout the range, undertake long migrations with populations extending to the headwaters of Yukon River over 2800 km upriver (Buklis and Barton 1984; Flannery *et al.* 2010). In addition, significant divergence in phenotypic and genetic characters between the two ecotypes suggests different evolutionary histories in the recent past (Seeb *et al.* 2011). Here we only examined the summer ecotype. Further study of individual migration timing including the fall and summer ecotypes in the Yukon River could determine whether there is an association between inversion type and return. We also recommend follow up work with whole-genome sequencing combined with individual metadata to better determine if this inversion is adaptive, and what genes and gene variants may be involved (*i.e.*, Pearse *et al.* 2019).

### Sex-determining region

Sex in salmon is genetically controlled (Devlin and Nagahama 2002) and is governed by the sex-determining gene *sdY* (Yano *et al.* 2013). The identity of sex chromosomes differ across salmonid species due to translocation of the sex-determining region (Davidson *et al.* 2009), and in Atlantic salmon *sdY* has been identified on three different chromosomes (Eisbrenner *et al.* 2014). In this study, we identified the sex-chromosome in chum salmon as LG15 which is orthologous to Ssa03, one of the chromosomes containing the *sdY* gene in Atlantic salmon. Interestingly, the homeolog of LG15 is orthologous to the sex chromosome in coho salmon, lake whitefish, and sockeye salmon (Sutherland *et al.* 2017) suggesting that sex genes were silenced or lost on separate homeologs between species. Discrepancies between genetic and phenotypic sex have been observed in previous studies and have been speculated to be due to spontaneous sex reversal (Nagler *et al.* 2001), non-functional Y-chromosomes due to *sdY* exon loss (Cavileer *et al.* 2015), or the possibility of autosomal modifiers that may interact with *sdY* (Eisbrenner *et al.* 2014). However, discrepancies may also be explained by technical errors including error in phenotypic sex assignment or errors in sample records. The populations in this study were sexed visually which can be unreliable, particularly if fish were caught before secondary sex characteristics were fully developed (Lozorie and McIntosh 2014). Despite this, overall concordance between phenotypic sex and sex determined by PCA cluster was high (90% overall and 99% in the Mulchatna collection) suggesting that this is the true sex determining region. Future studies of this genomic region should involve new collections of samples with careful attention to accurate phenotypic sex identification and pairing of phenotypic data and tissue samples.

## Conclusion

Examining genome-wide patterns of LD is an important tool for evolutionary analysis. Many studies explicitly exclude loci with LD; these may be missing important patterns of genomic variation. We identified a co-locating inversion and sex-determining region in chum salmon by performing network analysis on patterns of LD. The signal of these features was strong enough to drive PCA of the full dataset, resulting in false population structure. Attempting to identify and account for genomic features should be standard practice in genome-scale datasets.
